# New Approach to the Dispersion of Silica in the Natural Rubber Matrix by Grafting with Biobased Compounds: The Compatibilizing Effect of l‑Cysteine

**DOI:** 10.1021/acsomega.6c00726

**Published:** 2026-04-09

**Authors:** Agnieszka Kołodziejczak-Radzimska, Mirosława Prochoń, Dariusz M. Bieliński, Jakub Wręczycki, Mateusz Imiela, Teofil Jesionowski

**Affiliations:** † Poznan University of Technology, Faculty of Chemical Technology, Institute of Chemical Technology and Engineering, Berdychowo 4, 60-965 Poznan, Poland; ‡ Faculty of Chemistry, Institute of Polymer & Dye Technology, 49584 Lodz University of Technology, Stefanowskiego 12/16, 90-924 Lodz, Poland

## Abstract

Precipitated silica was subjected to a sustainable strategy of surface modification with l-cysteine or functional silanes: 3-aminopropyltriethoxysilane or 3-mercaptopropyltrimethoxysilane (as a reference way), and the effects were compared from the point of view of the degree of coverage and the number of functional groups present on the filler surface. Because the silane coupling agent requires relatively high doses, being additionally hazardous, flammable, susceptible to failure during storage, and has to be incorporated into the organic dispersants and prehydrolysis treatment, the application of more convenient and green modifiers is sought. Infrared spectra and thermogravimetric analysis confirm the effective grafting of silica surface, with the greatest extent observed in the case of l-cysteine. The presence of both amino and thiol groups in the amino acid molecule is likely to be responsible for its much greater degree of adsorption on the filler surface. A greater degree of modification of the silica surface with l-cysteine comes together with better compatibility of the amino acid with natural rubber (NR). Unlike silanes, l-cysteine does not seem to interfere with the sulfur vulcanization process. The proposed modification of silica resulted in an increase in the cross-link density of vulcanizates and a change in the cross-link structure. The replacement of silica-coupling agents with l-cysteine resulted in an improvement in the macrodispersion of filler, represented by the dispersion index and indicated by the Payne effect. The changes in the cross-link density and structure, as well as improved filler dispersion and unchanged bound rubber content, result in an increase in the tensile strength and elongation at break, but contrary to silanization, decreasing stiffness of the vulcanizates. The presented results confirm the possibility of replacing conventional silane compatibilizers with l-cysteine in rubber technology, opening a new direction of research on the application of cheap protein derivatives obtained from recycled natural products.

## Graphical abstract

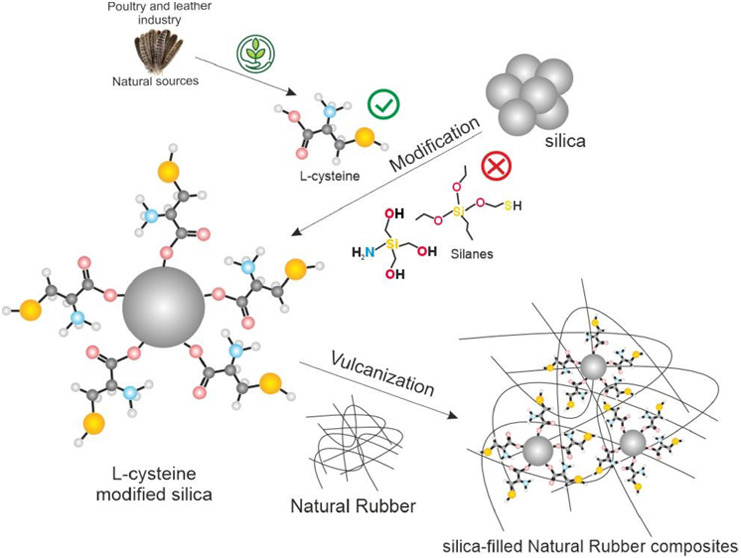

## 1 Introduction

The filler–rubber interaction is a key parameter for the reinforcement of the rubber. Silica is the most essential and cost-effective reinforcing filler in the rubber industry after carbon black. The silica reinforcement mechanism with a nonpolar elastomer, like natural rubber (NR), is complicated by the presence of polar functional groups on the filler surface. This polar nature of silica causes filler–filler interactions by forming hydrogen bonds. Therefore, sizable nondispersed silica clusters remain in a nonpolar rubber matrix.^1^ To avoid these strong filler–filler interactions and improve compatibility between rubber and silica, the filler surface must be modified. This can be done by using a coupling agent that has functional groups capable of linking both rubber and silica. Silanization is the most popular and effective covalent coating method for modifying the surface of a material rich in hydroxyl groups, such as silica or silicates.^2–9^ The mechanism of chemical reaction, improving the external filler–rubber interactions on the nanoscale by creating chemical links between the silica surface and the rubber chains, was described by Noordermeer and Dierkes.^10^ There are many types of commercially available silane coupling agents, which are easy to react with the hydroxylated surface and additionally introduce active groups (e.g., amino, thiol, and carboxyl groups) to the surface.^1,11–13^ Other methods developed to increase the dispersion of silica in the rubber matrix include the addition of a second filler^14^ or the improved interface structure.^15^ Nevertheless, the problem of the dispersion of silica in natural rubber, important for the tire industry,^16^ still remains unsolved.

The proteins present in NR exhibit a similar, although not as strong, effect as silane in hydrophobizing the silica surface.^17^ The Payne effect^18^ for NR compounds without silane decreases with the increasing content of proteins in the NR. The role of the proteins is to disrupt the silica–silica network, resulting in lower filler–filler interactions. This effect is not seen in the compounds containing silanized silica, as silica–silane coupling apparently is much stronger than the silica–protein interactions. Another possible explanation is the denaturation of protein after vulcanization, which results in the breakage of silica–protein interactions and consequently gives rise to a silica–silica network to reform.

The positive effect of the proteins present in NR on the dispersion of silica in rubber matrix has been translated into numerous patents, describing the treatment of silicates or silicas with an agent, possibly including an amino acid or polypeptide comprising an anhydride, a cyclic imide, and/or its derivatives, to be further applied in rubber mixtures.^19^ The compatibilization of high-surface materials with rubber matrix was also improved by the addition of amphiphilic hydrogel, containing, among others, collagen peptide and gelatin, to the rubber mixture.^20^

Because the silane coupling agent requires relatively high doses, being additionally hazardous, flammable, and susceptible to failure during storage, and has to be incorporated into the organic dispersants and prehydrolysis treatment, the application of more convenient and green modifiers is sought. The appropriate candidates seem to be amino acids containing sulfur, such as cysteine or methionine, of which l-cysteine, which possesses an active thiol group (–SH), looks especially promising.^21^ It is frequently employed in the surface modification of a variety of materials, including heavy-metal (hazardous) ion adsorbents,^22^ sensor materials for their detection,^23,24^ cancer-targeted drug carriers,^25,26^ metal epoxy anticorrosion coatings,^24^ etc., but there are few kinds of research on the application of l-cysteine as a modifier of rubber fillers. Duan et al. used l-cysteine to modify graphene or graphene oxide nanosheets^27^ to use them as fillers for natural rubber vulcanizates. Based on the change in the vulcanization curve of the rubber mixtures, he confirmed that the sulfhydryl group of l-cysteine could function as a cross-linking point to establish a strong chemical bond between the surface and the molecular chains of NR. Zhang et al.^28^ conducted the investigation of the reaction between the sulfhydryl groups of 3-mercaptopropyltrimethoxysilane-modified graphene oxide (GO) and the vinyl groups in the molecules of styrene–butadiene rubber (SBR). The results of attenuated total reflectance-Fourier-transform infrared spectroscopy (ATR-FTIR) analysis indicated that certain vinyl groups in SBR can react with the sulfhydryl groups of the modified GO during the cross-linking of rubber. Heinrich et al.^29^ pointed out the possibility of using l-cystine (cysteine dimer), or better its derivative, l-cystine,1,1′-dimethyl ester, hydrochloride (ELCH), as an effective secondary accelerator (along with CBS) of sulfur vulcanization of NR, allowing the elimination of 1,3-diphenylguanidine (DPG), the decomposition product of which, aniline, is toxic.^30,31^ DPG is officially classified in the EU (CLP Regulation) as toxic to aquatic life with long-lasting effects, harmful if swallowed, causing serious eye irritation, suspected of damaging fertility, and causing skin and respiratory irritation. Because of these hazards, companies increasingly reduce or replace DPG to meet workplace safety standards and reduce toxicological risks. There is also a possibility that DPG and aniline molecules, which generally accelerate sulfur vulcanization from a chemical point of view, may, on the other hand, obscure the silica surface from the second accelerator and its decomposition products, e.g., making its silanization more difficult, which could be avoided by using ELCH instead of DPG. In the presence of CBS, ELCH significantly accelerates the vulcanization process and increases the cross-link density of NR vulcanizates.^29^ Nevertheless, the substitution of DPG with l-cysteine or methionine slows down the vulcanization process in styrene–butadiene rubber compounds filled with silanized silica.^32^ When proteins containing significant amounts of sulfur amino acids were added to rubbers, an increase in the efficiency of the cross-linking process was observed.^33^ Encouraged by this fact, we decided to check whether, in addition to the possibility of eliminating DPG, it would also be possible to replace the silica silanization by modifying its surface with l-cysteine. In turn, the strengthening of mechanical properties is caused by, among others, the presence of numerous intermolecular interactions between the rubber matrix and the functional groups of the added proteins. Therefore, metal–cysteine coordination complexes were used to cross-link carboxylated butadiene-acrylonitrile rubber (XNBR), which allows its room-temperature self-healing and recyclability.^34^ The ability of waste keratin or its hydrolysates to modify synthetic rubbers was confirmed by our previous works.^33,35,36^ However, due to their high molecular weight and the lack of ability to modify the surface of silica,^37–40^ they were applied in the form of hybrid fillers with polymers and/or plasticizers.

As can be seen, l-cysteine with its active sulfhydryl group has the potential to serve as a surface modifier for inorganic rubber reinforcing fillers. The compatibility of the amino acid with natural rubber can also be an advantage. In addition, l-cysteine has more advantages than traditional coupling agents,^41^ including the possibility of recovery from protein waste (feathers and hair) from the poultry and leather industries, being environmentally friendly and biodegradable. Research on the use of protein hydrolysates for the modification of silica seems to be a natural continuation of the work involving the amino acid.

To the best of our knowledge, silica modified with l-cysteine was not used as a rubber filler. Based on that, this study compares l-cysteine applied as a green and low-cost surface modifier for precipitated silica with the ex situ silanization of the filler using functional silanes: 3-aminopropyltriethoxysilane or 3-mercaptopropyltrimethoxysilane, possessing amino and thiol groups, respectively. The degree of surface modification of silica was compared, and its influence on the filler macrodispersion, vulcanization characteristics, cross-link density, structure, and the resultant mechanical and dynamical properties of silica-filled NR vulcanizates was determined. In this work, the possibility of replacing the traditional coupling agent with a cheaper, more environmentally friendly alternative was looked into.

## 2 Experimental Section

### 2.1 Materials

#### 2.1.1 Modification of Silica Surface

Commercial precipitated silica Ebrosil H155 AT (IQESIL S.A., Zaragoza, Spain) of the Brunauer–Emmett–Teller (BET) surface area of 155 m^2^/g was subjected to surface treatment with l-cysteine (Pol-Aura Ltd., Zabrze, Poland) or functional silanes: 3-aminopropyltriethoxysilane (U 13, Unisil Ltd., Tarnów, Poland) or 3-mercaptopropyltrimethoxysilane (Dynasylan MTMO, Evonik Industries AG, Wesseling, Germany).

##### l-Cysteine Treatment

A 5% l-cysteine solution was prepared and mixed with silica for 24 h at room temperature (10 g of SiO_2_ and 100 cm^3^ of the l-cysteine solution). The system was filtered, and the modified silica was dried for 12 h at 105 °C.

##### Ex Situ Silanization

In the first step, the silane coupling agents were hydrolyzed with a 4:1 methanol/water solution. For the modification, 3 wt/wt of the appropriate silane was used. The silane solution was stirred with silica for 24 h at ambient temperature, being sufficient for the condensation reaction in MeOH. Before characterization, the modified silica was purified through washing and filtration to remove unreacted reagents, followed by removing traces of solvent, and final drying at 105 °C for 6 h of the chemically treated silica filler.

#### 2.1.2 Rubber Mixtures

The object of the research was rubber mixtures based on natural rubber grade RSS 3, filled with commercial precipitated silica Ebrosil H155 AT (see Table 1, native or previously subjected to surface modification with l-cysteine or selected silanes: either 3-aminopropyltriethoxysilane or 3-mercaptopropyltrimethoxysilane) to improve the degree of silica dispersion in the rubber and the interphase interactions. The other rubber compounding ingredients, zinc oxide, stearic acid, accelerator CBS (CZ, *N*-cyclohexyl-2-benzothiazole sulfonamide), and sulfur, were also provided by Torimex Group Ltd. The composition of the rubber mixtures studied deliberately excluded the addition of antiaging agents (NR is susceptible to degradation), e.g., *p*-phenylenediamine-based antiozonant *N*-isopropyl-*N*′-phenyl-1,4-phenylenediamine (IPPD) is usually used in conjunction with an amino antioxidant 2,2,4-trimethyl-1,2-dihydroquinoline (TMQ) due to the possible additional modification of the silica surface and their potential impact on the course of the vulcanization process. The results of the works by Noordermeer and colleagues highlight the strong influence of amines in silica-reinforced rubber compounds.^42,43^

**Table 1:** Composition of the Rubber Mixtures Studied

No.	components	content (phr)
1	natural rubber (NR)	100
2	stearic acid	2
3	zinc oxide	5
4	silica/modified silica	50
5	accelerator CBS	0.5
6	sulfur	2.5

The effect of compatibilization of silica with the NR matrix, obtained by the modification of the filler surface with an amino acid corresponding to their chemical structure, was compared with systems filled with native and amino- or mercaptosilane-modified silica-coupling compounds commonly used in rubber technology. These silanes were selected intentionally due to their chemical structure, containing chemical groups present in l-cysteine, although they are not the concensual choice for NR-based compounds.^44,45^ For this purpose, bifunctional organosilanes such as bis­(triethoxysilylpropyl) tetrasulfide (TESPT) or bis­(triethoxysilylpropyl) disulfide (TESPD) are commonly used in the tire industry. Blume and co-workers^46^ demonstrated that presilanization is more effective than in situ silanization during a two-stage mixing. This has additionally encouraged us to follow the former way of silica modification, despite economic drawbacks related to the time and cost of the ex situ treatment.

Rubber mixtures were prepared using a Brabender laboratory internal mixer (Germany) in four stages (Table 2):

- Stage 1: preparation of a rubber premix containing NR and 80 phr of native or modified silica (the increase of filler loading is a commonly used procedure to achieve greater shear stresses and improve the dispersion of the filler in rubber^47^)/fill factor 70%.
- Stage 2: incorporation of the remaining amount of rubber (diluting the rubber mixture to its final composition) and other ingredients into the premix prepared in Stage 1.
- Stage 3: incorporation of the cross-linking agents into the rubber masterbatch prepared in Stage 2.
- Stage 4: additional homogenization of all ingredients, followed by dumping of the mixture.

**Table 2:** Conditions for Preparing Rubber Mixtures

stage	stage description	time (min)	temperature (°C)	rotor speed (rpm)
1	rubber premix mastication	3	70	30
2	incorporation of remaining rubber, ZnO, and stearic acid	4	70	20
3	incorporation of CBS and sulfur	2	70	20
4	homogenization of ingredients	1	max. 75	40

#### 2.1.3 Rubber Vulcanizates

Rubber mixtures described above were vulcanized in appropriate steel molds at a temperature of 150 °C and a time *t*_90_, determined vulcametrically, according to ISO 3417.

### 2.2 Methods

#### 2.2.1 FT-IR Analysis of Silica Surface

In order to identify characteristic functional groups in the structure of the analyzed silica samples before and after the modification process, infrared analysis (FT-IR) was carried out using a Vertex 70 spectrometer from Bruker (Germany), equipped with an Mercuric Cadmium Telluride (MCT) detector. Before the analysis began, it was necessary to prepare a KBr pellet. For this purpose, 1.5 mg of the tested silica was ground in a mortar with 250 mg of anhydrous potassium bromide (Sigma-Aldrich). The mixture was pressed under a pressure of 10 MPa, and the obtained pellet was placed in the measuring chamber of the spectrometer. FT-IR analysis was performed in the wavenumber range of 4000–420 cm^–1^ with a resolution of 0.5 cm^–1^.

#### 2.2.2 NMR Measurement

^29^Si cross-polarization (CP) MAS spectra were recorded on a Bruker AV III 600 MHz spectrometer (Bruker GmBH Fallanden, Ettlingen, Germany). Spectra were acquired with a 4 mm magic-angle spinning (MAS) probe head by using ZrO_2_ rotors at a MAS frequency of 8 kHz. A proton 90° pulse length of 3.3 μs and a 9 ms contact pulse with a 50% ramp were applied. The repetition delay was 4 s, and the spectral width was 66.9 kHz. In total, 1024 scans were accumulated with a time domain size of 4 K data points and decoupling spinal 64 applied during acquisition (AQ = 30.5 ms).

#### 2.2.3 Thermogravimetry/Derivative TG (TG/DTG) Analysis of Silica

Thermogravimetric analysis (TG/DTG) was performed using a Jupiter STA 449 F3 instrument from Netzsch (Germany), enabling precise measurement of mass changes and thermal effects over a wide temperature range as a function of time. To carry out the measurement, it was necessary to prepare a sample weighing approximately 10 mg in an aluminum crucible, which was then burned in a nitrogen atmosphere in the temperature range of 30–1080 °C. The change in sample weight was recorded with a heating rate of 5 °C/min. Based on the data obtained, TG and DTG curves were plotted. The TG curve reproduces the change in mass as a function of temperature, thanks to which it is possible to observe the stages associated with the loss of mass of the sample during its heating. In turn, the DTG curve (thermogravimetric differential curve, the first derivative of the TG curve: d*m*/d*t* = *f*(*T*)) is a helpful tool in interpreting the results of TG analysis.

#### 2.2.4 Scanning Electron Microscopy (SEM) and Particle-Size Distribution (PSD) Analysis of the Unmodified and Modified Silica

The noninvasive backscattering method (NIBS) was used to determine the particle-size distribution (in the diameter range of 0.6–6000 nm) of the unmodified and modified silica. The Zetasizer Nano ZS apparatus (Malvern Instruments Ltd., Worcester, U.K.) was applied for this purpose. In order to visualize the morphology of the obtained inorganic fillers and hybrid materials, a scanning electron microscope-energy dispersive X-ray spectroscopy (SEM-EDS) Tescan MIRA3 (Princeton Gamma-Tech, Princeton, NJ) was used.

#### 2.2.5 Elemental Analysis and Degree of Coverage of Silica Surface

The subject of the analysis was the evaluation of the percentage of carbon, hydrogen, nitrogen, and sulfur in silica samples before and after surface modification. Measurements were carried out using a Vario EL Cube instrument from Elementar Analysensysteme GmbH (Germany). An important step of research before analysis was the appropriate preparation of samples. The 10 mg portion of an appropriate sample was closed in aluminum vessels. The sample went to a carousel, from which it was fed to the combustion column, where a controlled stream of oxygen caused the substance to burn at 1150 °C. The gases released in the process were directed to the reduction column, where they were transformed into the appropriate oxides and then adsorbed by dedicated adsorption columns. Using a detector, the amount of gas was analyzed and converted to the percentage composition of individual elements.

Based on the results obtained, the degree of coverage (*P*) and the number of functional groups (*N*_R_) present on the silica surface were calculated, using formulas [Disp-formula eq1] and 2:

1 $$P=\frac{10^{6}\times C}{\left[1200\times N_{c}-C\left(M-1\right)\right]\times A}$$

where *C* is the carbon content from elemental analysis, *N*_R_ is the number of carbons in the modifier molecule, *M* is the molar mass of the modifier, and *A* is the specific surface area of SiO_2_

and

2 $$N_{R}=\frac{C\times N_{A}}{12\times n\times 100\times S}$$

where *C* is the carbon content from elemental analysis, *N*_A_ is the Avogadro number, *n* is the number of carbons in the modifier molecule, including methoxy groups, and *S* is the specific surface area of the modified material.

#### 2.2.6 Kinetics of Vulcanization

The kinetics of vulcanization of rubber mixtures filled with silica were tested by using an oscillating MDR 3000 rheometer from Alpha Technologies, according to ISO 3417. During the measurement, the values of the following vulcanization parameters were determined: *M*_min_ is the minimum vulcametric torque, *M*_max_ is the maximum vulcametric torque, Δ*M* is the increase in vulcametric torque, *t*_02_ is the scorch time, and *t*_90_ is the optimal vulcanization time, and the cure rate index CRI = 100/(*t*_90_ – *t*_02_) is calculated.

#### 2.2.7 Cross-Link Density and Structure of Rubber Vulcanizates

The cross-link density of the filled vulcanizates was determined by the equilibrium swelling method in toluene. The 20–40 mg samples were placed in a weighing vessel and poured with solvent (toluene). The closed weighing vessels were placed in a laboratory heating chamber at 25 °C. After 48 h, the samples were weighed on an analytical balance, removing excess solvent. The samples were then dried to a constant weight in an oven at 60 °C.

The value of the equilibrium swelling of vulcanizates in toluene (*Q*_W_) was determined from formula [Disp-formula eq3]:

3 $$Q_{W}=\frac{\left[{(m}_{\mathrm{sp}}-m_{n}\right)-\left(m_{0}-m_{n}\right)]}{\left(m_{0}-m_{n}\right)}=\frac{\left(m_{\mathrm{sp}}-m_{n}\right)}{\left(m_{0}-m_{n}\right)}-1$$

where *Q*_W_ is the equilibrium swelling value, *m*_sp_ is the mass of the swollen sample, *m*_n_ is the mass of the filler in the vulcanizate, and *m*_0_ is the initial weight of the “dry” sample, before placing it in the solvent.

Equilibrium volumetric swelling of vulcanizates (*Q*_v_) was calculated from formula [Disp-formula eq4]:

4 $$Q_{v}=Q_{w}\frac{d_{k}}{d_{r}}$$

where *d*_k_ is the rubber density and *d*_r_ is the solvent density (*d*_r_ = 0.867 g/cm^3^ for toluene).

Based on *Q*_v_, the volume fraction of rubber in the swollen sample (*V*_r_) was calculated (formula [Disp-formula eq5]):

5 $$V_{r}=\frac{1}{{1+Q}_{v}}$$

which was used to calculate the cross-link density of vulcanizates (ν), using the Flory–Rehner eq 6:^48^

6 $$\nu =\frac{-[\ln\left(1-V_{r}\right)+V_{r}+\mu V_{r}^{2}]}{V_{0}\cdot (V_{r}^{1/3}-\frac{2V_{r}}{f})}$$

where *f* is the network functionality (*f* = 4), *V*_0_ is the molar volume of solvent (106.2 mL/mol for toluene), and μ is the Huggins parameter of cross-linked rubber–solvent interactions (μ = 0.385 for the NR–toluene couple).

The cross-link structure of vulcanizates was determined by thiol-amine analysis (OTAM-OTAT).^49^ The determinations involved the selective uncoupling of appropriate bridges by solvents and then subsequent swelling of the material, enabling the determination of the amount of a given remaining type of cross-links in the vulcanizate. Soft thiol-amine reagent (OTAM), a solution of isopropyl mercaptan (0.4 M) and piperidine (0.4 M) in toluene, causes the cleavage of polysulfidic bridges. Rubber samples weighing approximately 200 mg were then exposed to the OTAM reagent for 3 h in a nitrogen atmosphere. Hard thiol-amine reagent (OTAT), a solution of dodecyl mercaptan (1.0 M) in piperidine, causes the cleavage of disulfidic and polysulfidic bridges. Rubber samples weighing approximately 200 mg were exposed to the OTAT solution for 24 h in a nitrogen atmosphere. Each time after exposure to the above-mentioned solvents, the samples were washed with toluene and then swelled in toluene for 72 h. After drying on filter paper, the swollen samples were weighed and then placed in a Binder laboratory dryer (Germany) to be weighed again after drying to constant weight (72 h/60 °C).

Based on the data obtained, calculations were made of the cross-link density of vulcanizates treated with OTAM and OTAT solutions, according to formulas [Disp-formula eq3]–[Disp-formula eq6]. The shares of specific cross-links were determined on the basis of

- monosulfidic and C–C cross-links, the result of equilibrium swelling of vulcanizates in toluene after the action of a hard OTAT reagent,
- disulfidic cross-links, from the difference in the amount of equilibrium swelling of vulcanizates in toluene treated with a soft OTAM reagent and a hard OTAT reagent, and
- polysulfidic cross-links, as the difference in swelling of vulcanizates in toluene without and after the action of a soft OTAM reagent.

#### 2.2.8 Bound Rubber Content (BdR)

The content of bound rubber (BdR) in the rubber mixtures studied was determined on the basis of the weight loss of the samples (not cross-linked) with an initial weight of approximately 0.5 g, wrapped in a filter paper. Each of them was placed in a vessel containing 100 mL of toluene for 7 days at room temperature. The solvent was replaced with a new one after 4 days. In the end, the samples were dried to a constant weight in a vacuum dryer and weighed again. The percentage of rubber bound in rubber mixtures (BdR%) was calculated using formula [Disp-formula eq7]:

7 $$\mathrm{BdR}\%=\frac{M_{B}-M_{F}-M_{D}}{M_{B}}\times 100\%$$

where *M*_B_ is the mass of the sample of rubber mixture, *M*_F_ is the mass of filler in the sample, and *M*_D_ is the mass of the sample swollen in toluene.

#### 2.2.9 Morphology of Rubber Vulcanizates

##### 2.2.9.1 SEM-EDS

The micromorphology of the rubber vulcanizates studied was examined in their cross sections using S-4700, an field emission (FE) scanning electron microscope from Hitachi (Japan), equipped with an EDS detector from ThermoScientific. Samples covered with an Au layer, using a Cressington 208 HRD sputter (U.K.), were placed on an aluminum SEM stage. The measurements were performed at a current that accelerates the electron beam of 25 kV, in analysis mode, at 500- and 200-times magnification. To analyze the degree of silica dispersion in vulcanizate samples, EDS maps of their cross sections were made.

##### 2.2.9.2 Degree of Filler Dispersion (DI)

The influence of silica modification on macrodispersion of the filler in NR vulcanizates was studied with a MonTech DisperTester 3000 (Columbia City, IN). The test method, based on the ISO 11345 standard, quantifies the surface roughness of a freshly cut specimen using an optical microscope operating in the reflection mode.

#### 2.2.10 Payne Effect

The Payne effect^18^ for the vulcanizates studied was determined using a MonTech D-RPA 3000 instrument (Columbia City, IN), operating in the deformation range of 0.5–100% at a temperature of 100 °C. The Payne effect of the rubber mixtures was determined as the difference between the value of the mechanical modulus at the largest (100%) and the smallest (0.56%) relative sample deformation.

#### 2.2.11 Dynamic Mechanical Thermal Analysis (DMTA) of Rubber Vulcanizates

The dynamic properties of the rubber vulcanizates studied were determined by applying a dynamic thermomechanical analysis (DMTA) method using a Mettler Toledo DMA/SDTA861e analyzer (Columbus, OH) operating in the tensile mode. Measurements of elastic parameters (storage modulus, *E*′; loss modulus, *E″*), as well as the tangent of the loss angle (tan δ), were carried out in the temperature range from −120 to 80 °C with a heating rate of 3 °C/min, a given strain amplitude of 4 μm, and a frequency of 1 Hz. The glass transition temperature of the rubber vulcanizates was determined at the point where the maximum value of the function tan δ = *f*(*T*) occurred.

#### 2.2.12 Mechanical Properties of Rubber Vulcanizates

The mechanical properties of the rubber vulcanizates studied under static stretching conditions were determined by using a ZwickRoell 1435 universal testing machine, cooperating with an optical extensometer (Ulm, Germany), in accordance with the ISO 37 standard. The tests were carried out on type 3 dumbbell specimens. The mechanical modules of the vulcanizates were determined with relative elongation of 100, 200, and 300%, together with tensile strength, TS, and elongation at break, *E*_b_, of the samples.

The hardness of the rubber vulcanizates studied was determined with a ZwickRoell Shore A 3130 hardness tester (Ulm, Germany) in accordance with the PN-EN ISO 868 standard.

## 3 Results and Discussion

### 3.1 FT-IR and NMR Analysis of Silica Surface

The normalized FT-IR spectra of pure and modified silica are shown in Figure 1. Important structural features of native silica in its infrared spectrum (blue line) include the wide peak in the range of 1020–1250 cm^–1^, attributed to Si–O–Si bonds, and the peaks in the range of 3468, 1639, and 1420 cm^–1^, related to the stretching (two first) and bending (the last one) vibrations of the OH groups. The appearance of two peaks at 2925 and 2850 cm^–1^ (Figure 1d) for the amino-, mercapto-, and cysteine-modified silica is related to the symmetric and asymmetric vibrations in the CH_2_ groups belonging to the silane and cysteine, and it is a clear confirmation of the initial surface modification of silica.^50^ In addition, the intensification of the bands at approximately 3500 and 1200–1035 cm^–1^ (Figure 1b) can confirm the chemisorption of the modifiers on the silica surface. Apart from these, in the FT-IR spectrum of SiO_2__aminosilane (Figure 1d, red line), the characteristic peak of the N–H bond occurs at the wavenumber of 1512 cm^–1^, assigned to the deformation modes of the free amino groups.^51^ Furthermore, in the FT-IR spectrum of SiO_2__mercapto (Figure 1d, gray line), the peak at 1464 cm^–1^, corresponding to the Si–CH_2_ bending vibration present in organosilane, is observed. In addition, the SH corresponding to the grafting mercaptosilane molecules cannot be clearly observed in the spectra of modified silica (Figure 1a) because of the strong vibration of nanosilica, which could overlap the vibrations of the functional groups of 3-mercaptopropyltrimethoxysilane.^52,53^ In the FT-IR spectrum of SiO_2__l-cysteine (Figure 1a, green line), the bands at 2580 cm^–1^ correspond to the S–H stretching in cysteine, which indicates that the sulfur atom is not bound to silanol groups. Also, the band at 1483 cm^–1^ appears, which is attributed to the amino groups strongly hydrogen-bonded to free silanols. In addition, the intensification of the broad double band at 1628 and 1584 cm^–1^ is assigned to the asymmetric stretching vibration of COO–,^54^ which can also be overlaid with OH bending.^51,55,56^

**Figure 1:**
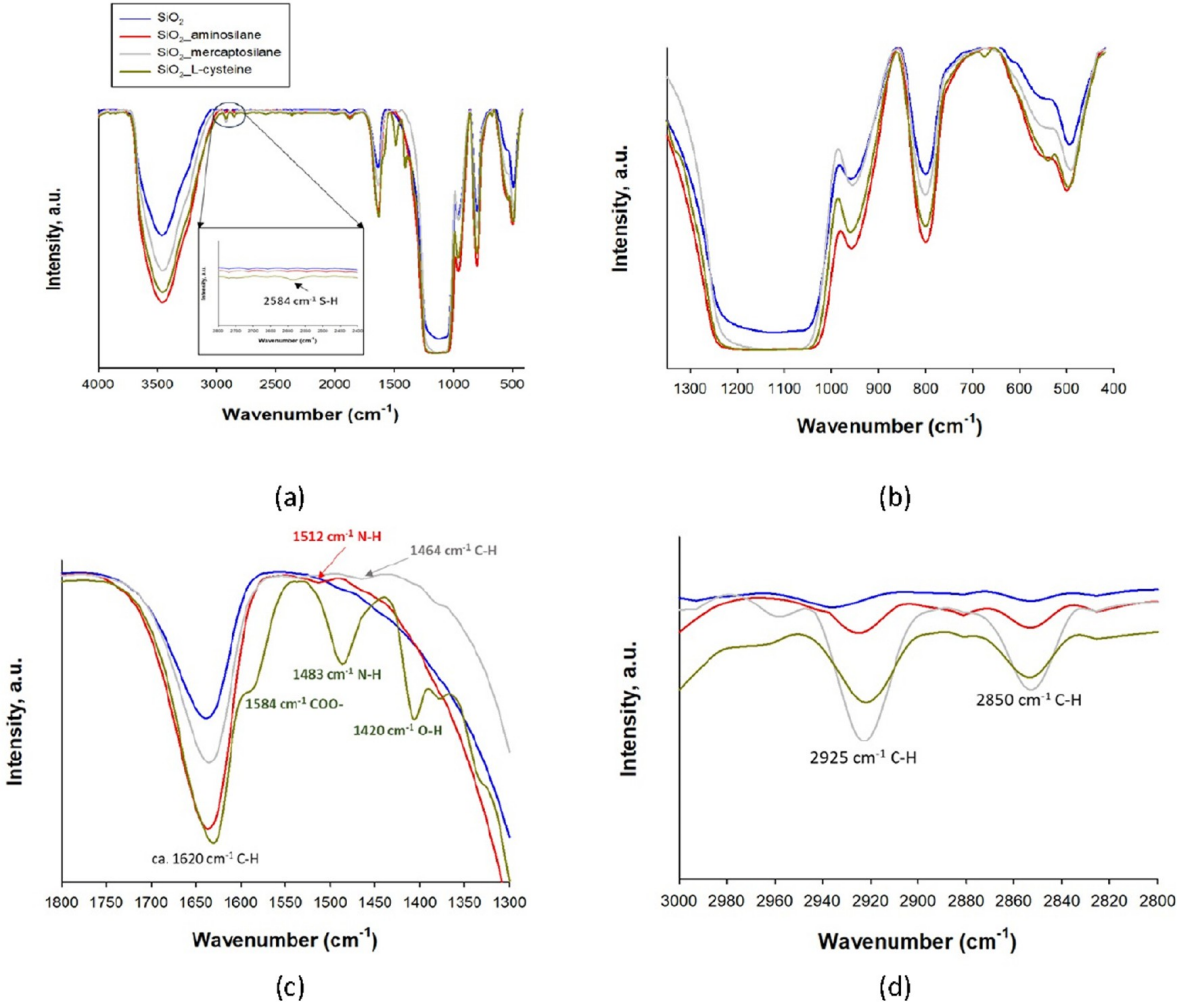
(a) Normalized FT-IR spectra of SiO_2__aminosilane, SiO_2__mercaptosilane, and SiO_2__l-cysteine compared to the spectrum of the native filler (SiO_2_). Infrared spectra in the different ranges of wavenumbers (b) 1300–400 cm^–1^, (c) 3000–2800 cm^–1^, and (d) 1300–1800 cm^–1^.

The solid-state ^29^Si NMR spectrum of the silica surface (for all samples) exhibited three peaks as shown in Figure 2: −90, −101, and −110 ppm. These peaks are attributed to pure surface signals and are assigned, respectively, to (HO)_2_Si­(OSi)_4_, HO-Si-(OSi)_3_, and Si­(OSi)_4_, accordingly corresponding to the Q_2_, Q_3_, and Q_4_ signals.^57^ Based on the presented NMR spectra, an increase in the intensity of the Q_4_ signal originating from siloxane groups (Si­(OSi)_4_) can be observed, which confirms the modification with silanes. The chemisorption of silane and the effectiveness of modification were confirmed by the detection of the T^2^ (57 nm) and T^3^ (68 nm) structures. The T^2^ corresponds to the silicon atom to the silylating agent bound to one −OH group forming the RSi­(OSi)­(OH)_2_, whereas T^3^ is assigned to RSi­(OSi)_3_.^58,59^ In the case of modification with cysteine, this is not observed as a result of the different types of this modifier.

**Figure 2:**
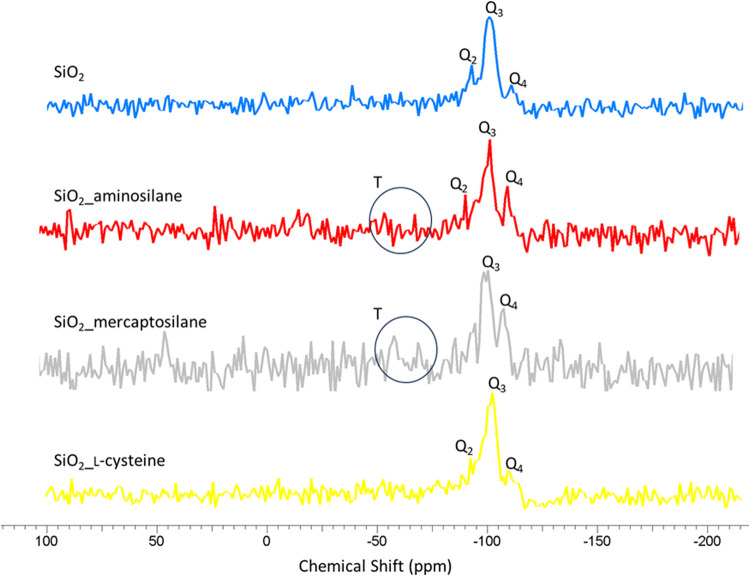
^29^Si CP MAS NMR spectra of unmodified and modified silica.

### 3.2 TG/DTG Analysis of Silica

In addition, the thermogravimetric curves for silane-modified silica do not differ much from the TGA curves for native silica (Figure 3). The mass loss is observed at ca. 88–90 °C related to physically adsorbed water. For three samples, SiO_2_, SiO_2__aminosilane, and SiO_2__mercaptosilane, a similar total mass loss (approximately 8.8, 8.5, and 8.4%, respectively) is observed.

**Figure 3:**
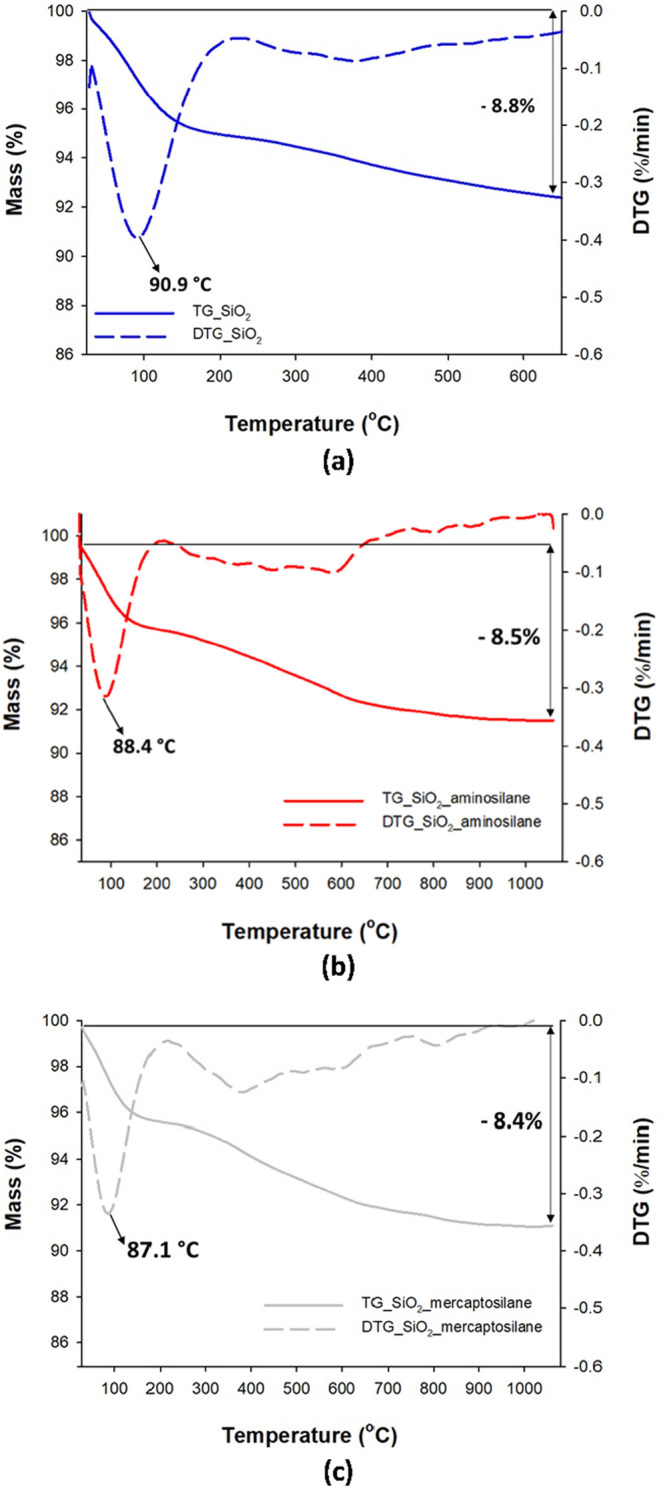
Thermogravimetric curves (TG and DTG) for (a) silica, (b) SiO_2__aminosilane, and (c) SiO_2__mercaptosilane.

However, in the case of the TG curves for silica modified with l-cysteine (Figure 4), we observe a greater weight loss (approximately 12%) compared to that of native silica, which can be attributed to the covering of silica with l-cysteine. Additionally, the DTG curve (Figure 4a) shows an exothermic peak at the same temperature as the peak on the DTG curve corresponding to pure l-cysteine (Figure 4b), in agreement with the literature.^60^

**Figure 4:**
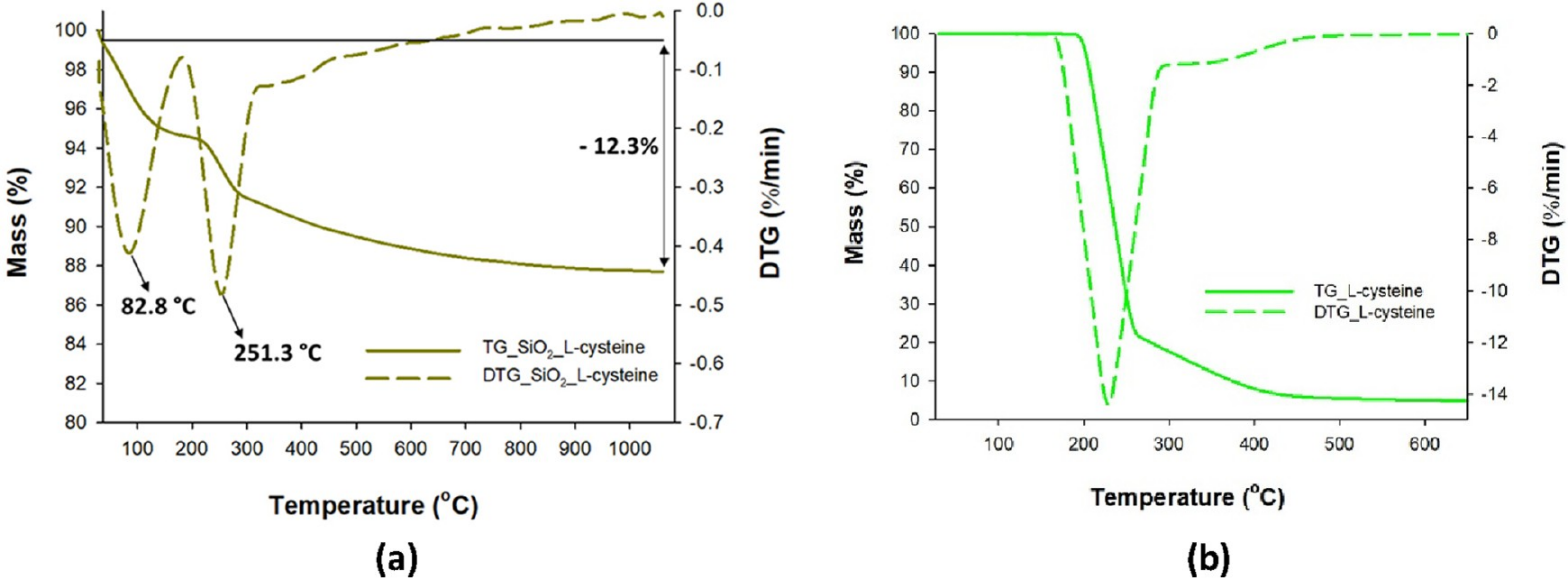
Thermogravimetric curves of TG/DTG for (a) l-cysteine-modified silica and (b) pure l-cysteine.

### 3.3 SEM and PSD Analysis of Both Unmodified and Modified Silica

To evaluate the particle agglomeration changes and particle-size distribution of the tested silica-based fillers after modification, the SEM and NIBS analyses were done. The obtained results are presented in Table 3. Native silica is characterized by a monomodal particle-size distribution with a relatively narrow diameter range of 122–342 nm. For SiO_2_, the maximum volume fraction is 28.1% and corresponds to particles with a diameter of 220 nm. In the case of silane-modified silica, a slight shift in the particle-size distribution toward smaller values can be observed: 142–342 nm for SiO_2_-aminosilane and 142–295 nm for SiO_2__mercaptosilane. In the case of SiO_2__aminosilane, the dominant fraction (28.6%) is characteristic of particles with a diameter of 220 nm, while for SiO_2__mercaptosilane, it is 33.8% (190 nm). However, in the case of SiO_2__l-cysteine, a more significant shift in the particle-size distribution toward smaller values can be observed. This sample is characterized by diameters in the range of 106–255 nm, with particles diameter of 164 nm (27.7%) dominating. In addition, SEM images of both unmodified and modified silica indicate the presence of particles that tend to aggregate into irregular shapes.

**Table 3:**
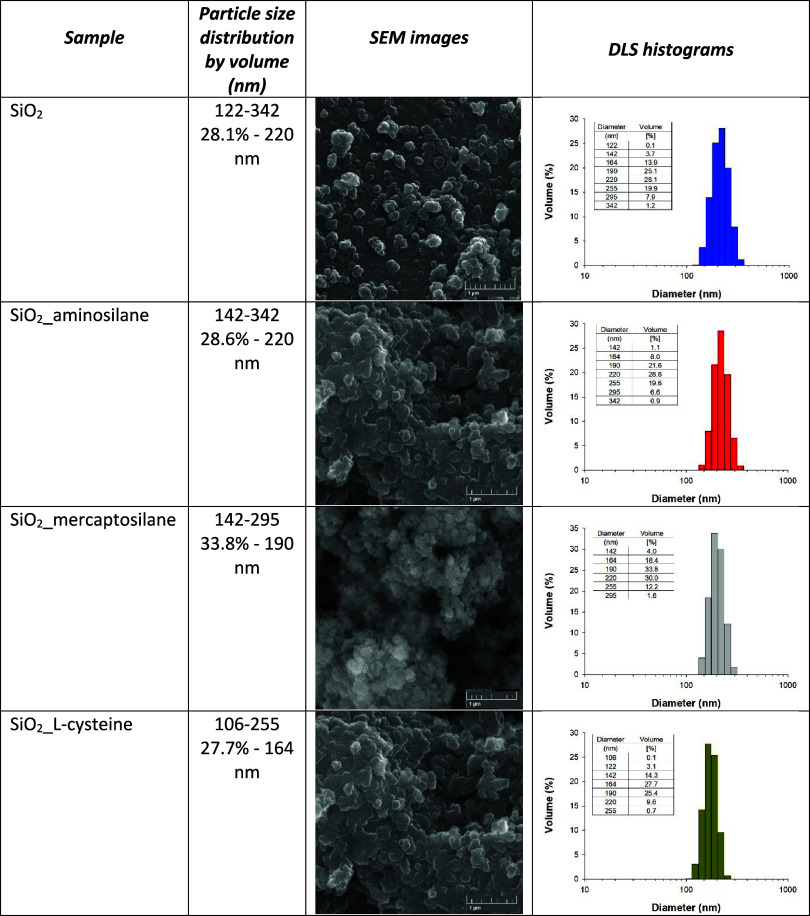
Dispersive and Morphological Properties of Both Unmodified and Modified Silica

### 3.4 Degree of Modification of the Silica Surface

Based on the results of the elemental analysis (Table 4), an increase in the content of individual elements can be observed in the silica samples after their surfaces were modified with both silanes and l-cysteine. In the case of modification with aminosilane, the nitrogen and carbon contents increased, while for the sample modified with mercaptosilane, an increase in carbon and sulfur content was observed. However, for the sample modified with l-cysteine, the contents of nitrogen, carbon, and sulfur increased. The changes in the elemental composition of the surface of the silica samples confirm the modification process.

**Table 4:** Elemental Analysis of the Surface of Silica Samples^a^

	elemental content (%)			
samples	N	C	H	S
SiO_2_	0.256	0.165	0.869	0.292
SiO_2__aminosilane	0.489	0.856	0.911	0.264
SiO_2__mercaptosilane	0.260	0.765	0.891	0.597
SiO_2__l-cysteine	0.414	1.127	1.456	1.068

a Weight content of nitrogen, carbon, hydrogen, and sulfur.

The degree of coverage with the appropriate modifier and the number of functional groups applied to the surface of the filler are presented in Table 5.

**Table 5:** Degree of Coverage (*P*) and the Number of Functional Groups (*N*_R_) on the Modified Silica Surface

samples	degree of coverage *P* (mmol/m^2^)	number of functional groups *N*_R_ (1/nm^2^)
SiO_2__aminosilane	0.85	0.10
SiO_2__mercaptosilane	0.75	0.09
SiO_2__l-cysteine	2.297	0.40

In the case of samples modified with silanes, a similar degree of coverage and number of functional groups on the silica surface was observed, while the degree of coverage and the number of groups derived from l-cysteine were higher. This seems rational, considering the fact that cysteine contains both NH and SH groups, and referring to these groups, the degree of coverage of the silica surface with them should be greater.

### 3.5 Kinetics and Parameters of Vulcanization

The vulcanization parameters of the rubber mixtures studied, determined on the basis of vulcametric curves (Figure 5), are given in Table 6.

**Figure 5:**
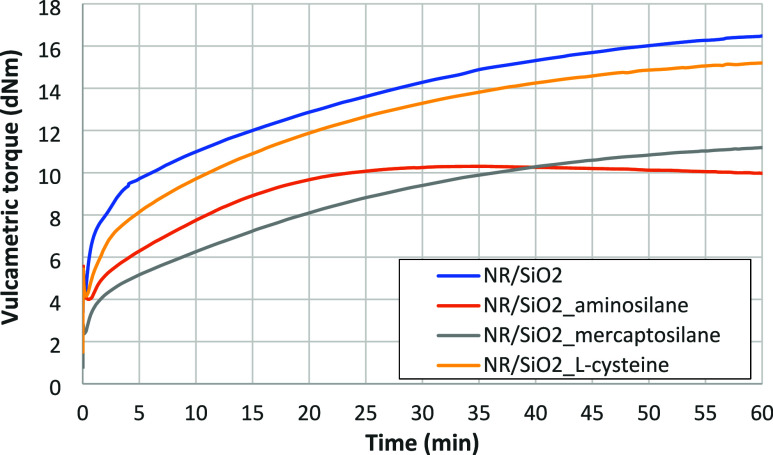
Vulcanization kinetics of the rubber mixtures filled with silica and modified silica.

**Table 6:** Vulcanization Parameters of the Rubber Mixtures Filled with Silica and Modified Silica

rubber mixtures	*M*_max_ (dNm)	*M*_min_ (dNm)	Δ*M* (dNm)	*M*_90_ (dNm)	*t*_90_ (min)	*t*_02_ (min)	CRI (min^–1^)
NR/SiO_2_	16.49	1.72	14.77	14.84	35.20	0.02	2.84
NR/SiO_2__aminosilane	10.30	1.32	8.98	9.27	17.00	0.01	5.89
NR/SiO_2__mercaptosilane	11.19	0.74	10.45	10.07	37.00	0.01	2.70
NR/SiO_2__l-cysteine	15.20	1.49	13.71	13.68	33.00	0.02	3.03

The kinetics of vulcanization (Figure 5) generally confirm the result obtained by Akikusa and Ito,^61^ who found that the scorch time of the sulfur vulcanization reaction of isoprene rubber (IR) was shortened by the addition of silica. The authors associate it with a decomposition of CBS by silanol groups of silica, which can be explained by the decomposition of the accelerator by the acid-catalyst ability of silanol groups. These results indicated that at an initial stage of vulcanization, most CBS was decomposed by silanol groups, which were covered with the adsorbed water, leading to the fast vulcanization reaction by sulfur. Unlike the silanes used, l-cysteine does not seem to change filler–rubber interactions, keeping its surface polar, thus interfering with the sulfur vulcanization process similar to native silica, achieving a similar increase in vulcametric torque. Nevertheless, the effect of a higher degree of modification of the silica surface with l-cysteine than with silanes (Table 5) and the better compatibility of the amino acid with natural rubber^40^ cannot be excluded. Also, finally, better vulcanization efficiency, measured by Δ*M* (Table 6), demonstrated by l-cysteine in comparison to silanes, can also be explained by its ability to accelerate sulfur vulcanization, as demonstrated by Heinrich and co-workers^29^ for l-cysteine derivative as an effective secondary accelerator (along with CBS) of sulfur vulcanization of natural rubber. However, taking into consideration filler–rubber interactions, represented by bound rubber values, being the highest for the NR-silanized silica mixtures studied (Table 7), and suppressing the amount of water molecules absorbed directly on the silica surface, the former explanation seems to be the most probable.

**Table 7:** Cross-Link Structure of Rubber Vulcanizates Filled with Native and Modified Silica^a^

	cross-link structure [%]		
samples	monosulfidic and S–S	disulfidic	polysulfidic
NR/SiO_2_	0	50	50
NR/SiO_2__mercaptosilane	dtbd	>30	64
NR/SiO_2__aminosilane	dtbd	dtbd	∼100
NR/SiO_2__l-cysteine	dtbd	dtbd	∼100

a dtbd: difficult to be determined.

### 3.6 Cross-Link Density and Structure of Rubber Vulcanizates

The cross-link density of vulcanizates filled with native silica and its variants modified on the surface is shown in Figure 6. Each modification, especially in relation to silanization, resulted in an increase in the cross-link density of the vulcanizates.

**Figure 6:**
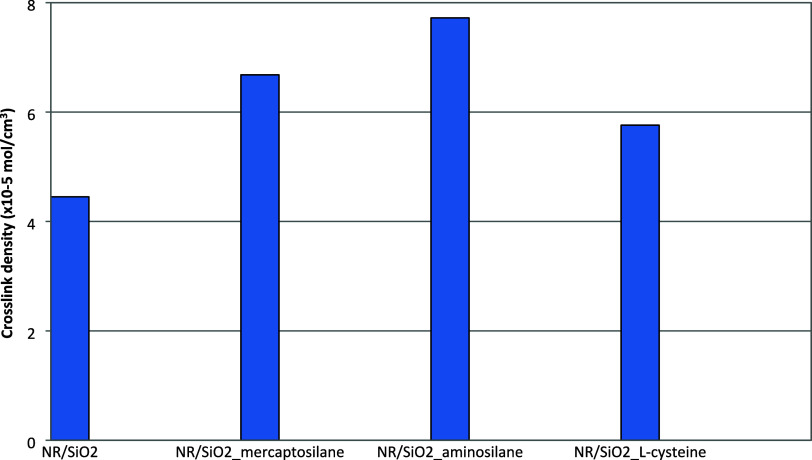
Cross-link density of rubber vulcanizates filled with native and modified silica.

The vulcanizate with native silica and the vulcanizates filled with silica modified with aminosilane and l-cysteine did not survive the test with either the “soft” reagent (OTAM), which selectively decomposes polysulfidic cross-links, or with the “hard” reagent (OTAT), which decomposes both polysulfidic and disulfidic cross-links. In these cases, the results obtained prove the almost exclusive presence of long sulfidic bridges between rubber macromolecules. In the case of the vulcanizate filled with silica modified with mercaptosilane, the density of the spatial network after selective decomposition of polysulfidic bonds was 2.40 × 10^–5^ mol/cm^3^. Calculated in relation to the initial value (6.68 × 10^–5^ mol/cm^3^), this indicates approximately 64% share of polysulfidic cross-links in the spatial network. However, this vulcanizate did not survive the analysis that decomposed poly- and disulfidic cross-links, leaving monosulfidic and carbon–carbon bridges in the network structure. This indicates a very low share of short cross-links (monosulfidic and C–C) and may indicate, by deduction, the share of disulfidic bonds at over 30%, which, however, cannot be determined directly using the thiol-amine analysis. The respective fractions of mono-, di-, and polysulfide cross-links of the studied vulcanizates are presented in Table 7.

### 3.7 Interactions at the Rubber–Silica Interface (BdR)

Table 8 lists the results of determining the BdR content in masterbatches containing 100 g of NR and 80 g of native or modified silica (approximately 44.5% by weight). The lowest content of bound rubber was characteristic of the masterbatch containing the l-cysteine-modified silica, which is at a level similar to the value for native silica. According to Noordermeer and co-workers, the silica–silica network can be disrupted even by proteins contained in NR.^17^ In this case, they act more like a surface covering agent. Silanization of silica with aminosilane or mercaptosilane significantly increased the amount of rubber unavailable to the extracting solvent.

**Table 8:** Bound Rubber (BdR) Content in the Rubber Mixtures Studied

samples	bound rubber content BdR (%)
NR/SiO_2_	19.2
NR/SiO_2__mercaptosilane	32.2
NR/SiO_2__aminosilane	34.8
NR/SiO_2__l-cysteine	22.9

### 3.8 Morphology of Rubber Vulcanizates

Si distribution maps in the vulcanizates studied indicate no significant differences in the degree of silica dispersion in microscale (Figure 7).

**Figure 7:**
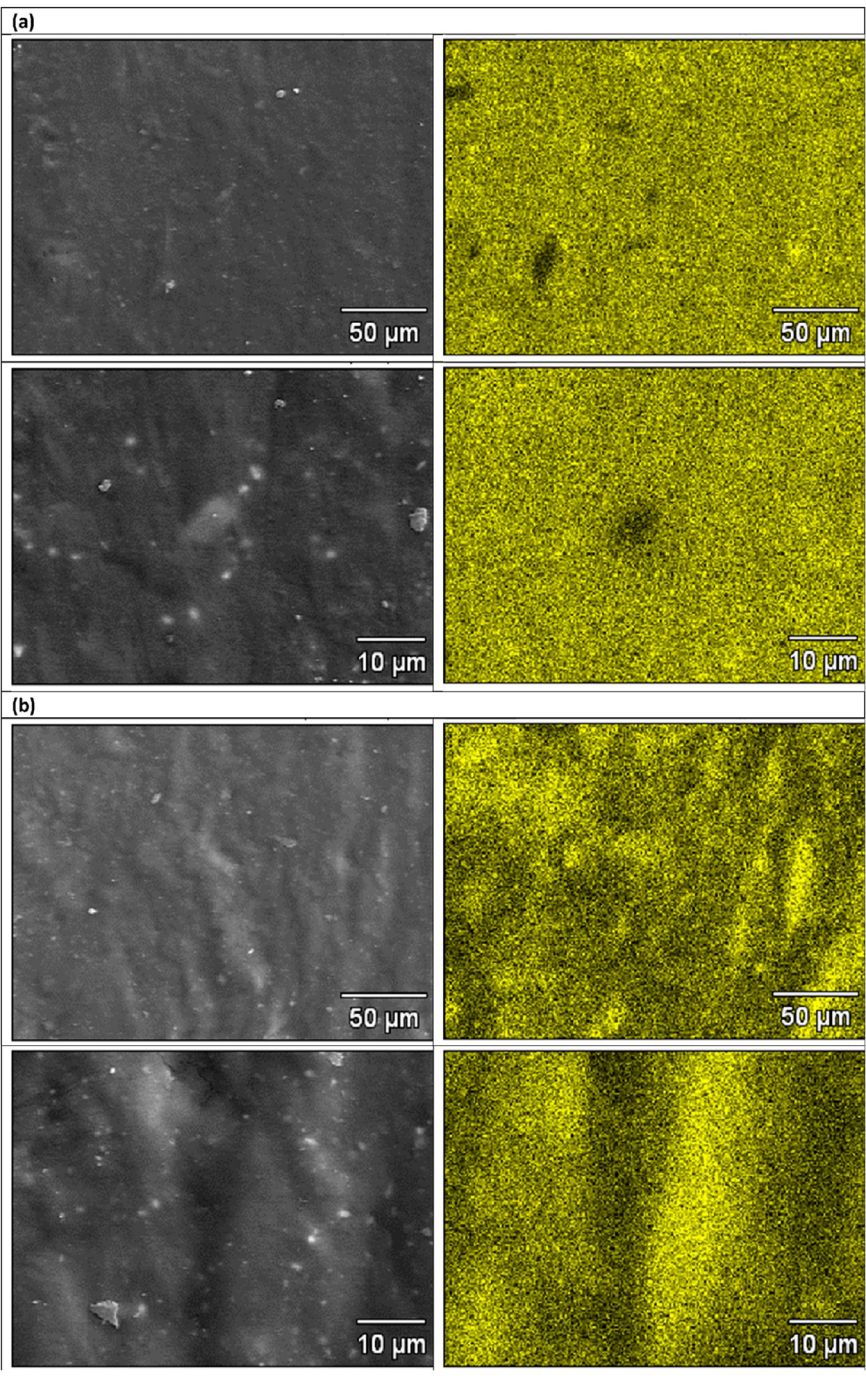

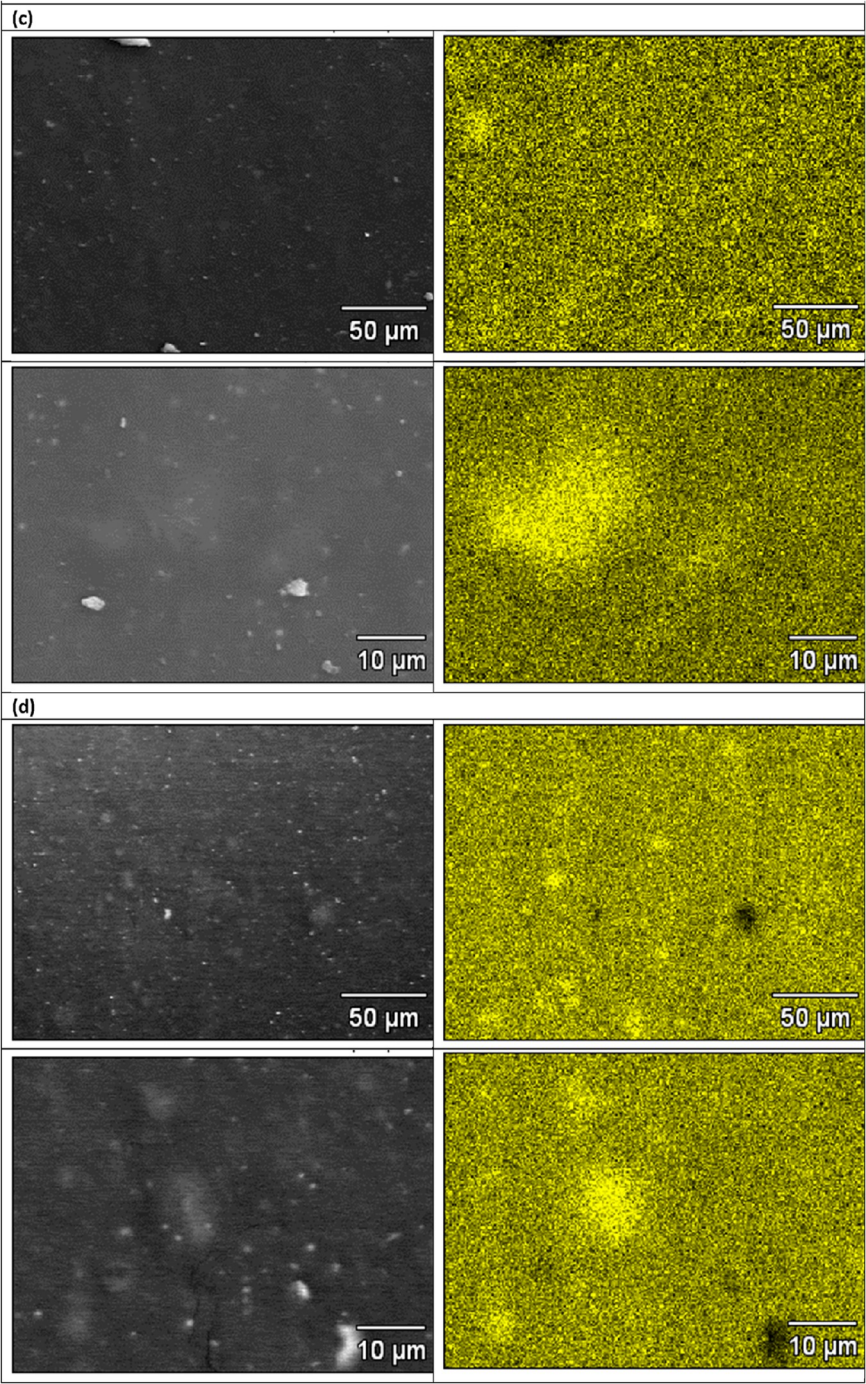
SEM images (left) and SEM/EDS maps of Si distribution (right) in the rubber vulcanizates studied for (a) NR/SiO_2_, (b) NR/SiO_2__aminosilane, (c) NR/SiO_2__mercaptosilane, and (d) NR/SiO_2__l-cysteine samples.

Apart from qualitative microanalysis, the influence of silica modification on the filler dispersion in the natural rubber matrix was quantified on a macroscale using the dispersion index (DI) (Table 9), containing DisperGrader data.

**Table 9:** Quantitative Analysis (DI) of the Influence of Silica Modification on Its Dispersion in NR

sample	dispersion (%)	average agglomerate size (μm)
NR/SiO_2_	53.96	3.89 ± 2.74
NR/SiO_2__aminosilane	74.84	4.17 ± 3.14
NR/SiO_2__mercaptosilane	68.19	3.54 ± 2.35
NR/SiO_2__l-cysteine	65.42	3.45 ± 2.28

The applied methodology of preparing rubber mixtures in several stages enabled obtaining good dispersion of silica in the rubber matrix.^47^ Filler–filler interactions or filler morphology is one of the major factors contributing to the bound rubber formation for untreated silica filler. The use of surface modification of the filler with mercaptosilane or l-cysteine slightly improved the microdispersion of silica in rubber, while the use of aminosilane slightly worsened it (Figure 7). DisperGrader data (Table 9) also demonstrate the positive influence of modifications on the macrodispersion of silica in a rubber matrix. However, the highest values of dispersion obtained for the NR/SiO_2__aminosilane sample are in agreement with the largest size of silica agglomerates, which can be the result of the condensation of the silane on silica particles and remain in agreement with elemental analysis of the surface of silica samples (Table 4). However, it should not be forgotten that the vulcametric parameters of rubber mixtures and the corresponding mechanical properties of vulcanizates are influenced not only by the dispersion of the filler but also by the dispersion of the components of the curing system,^62^ which may partially explain the results of vulcametric tests (Figure 5).

A certain modification toward the share of shorter cross-links in the case of silica modification with silanes (mostly mercaptosilane), and an increase in the cross-link density of the vulcanizates filled with modified silica, should translate into internal interactions in the filler phase (Payne effect) and the mechanical strength of the vulcanizates.

### 3.9 Internal Interactions in the Filler Phase: The Payne Effect

The hydrophobation of the silica surface by silane coupling agents reduces the filler–filler interaction by creating covalent bonds between the rubber and fillers. The greatest effect of weakening internal interactions in the filler phase was achieved by using mercaptosilane to modify silica, which most effectively (the smallest thickness of the adsorbed layer; Tables 3–5 and Figure 7) modified the silica surface while significantly increasing the interactions with the rubber matrix (Table 8). The effect of l-cysteine on silica–silica interaction stays in agreement with the results reported by Noordermeer and co-workers,^17^ who studied the influence of protein content in NR on the Payne effect. The proteins in NR were shown to have a similar effect as silane in hydrophobizing the silica surfaces, although not as strong.

The internal interactions in the filler phase are illustrated by the results of determining the Payne effect in the vulcanizates studied (Figure 8 and Table 10).

**Figure 8:**
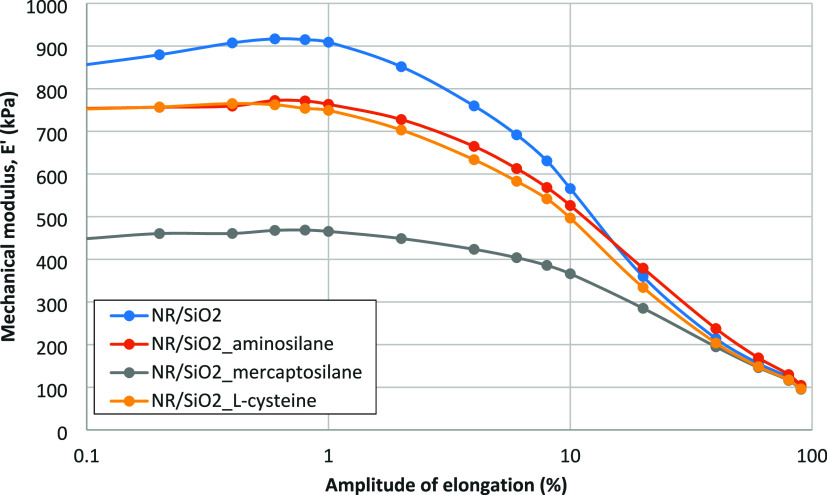
Payne effect for the rubber vulcanizates filled with both native and modified silica.

**Table 10:** Δ*E*′ Values Corresponding to the Payne Effect, Illustrated in Figure 8

samples	Δ*E*′ (kPa)
NR/SiO_2_	815.7
NR/SiO_2__mercaptosilane	372.6
NR/SiO_2__aminosilane	667.9
NR/SiO_2__l-cysteine	666.0

The decrease in internal filler–filler interactions, reflected by a lower Payne effect, correlates with an improvement in silica microdispersion, which is most pronounced for silica modified with mercaptosilane and l-cysteine (Table 9 and Figure 7), which indicates an improved macroscopic distribution of the filler. However, this is accompanied by an increase in the average agglomerate size (Table 9), i.e., a deterioration of silica microdispersion, which may adversely affect the mechanical properties of the filled rubber vulcanizates. In the case of modification of silica with aminosilane, the share of bound rubber is the highest, although comparable to mixtures filled with SiO_2__mercaptosilane, but in the first case, most likely, part of the modifier participates in the sulfur cross-linking reaction as a coagent. This is evidenced by the highest cross-link density of the vulcanizates in this case, confirmed by the results of mechanical tests under dynamic (Table 11) and static (Table 12) conditions.

**Table 11:** Results of DMTA Studies of Rubber Vulcanizates Filled with Silica

	parameters			
samples	*T*_g_ (°C)	tan δ_TG_	tan δ_0_	tan δ_60_
NR/SiO_2_	–72.5	1.09	0.09	0.13
NR/SiO_2__aminosilane	–68.9	1.07	0.08	0.11
NR/SiO_2__mercaptosilane	–71.9	1.25	0.12	0.14
NR/SiO_2__l-cysteine	–72.1	1.28	0.11	0.15

**Table 12:** Mechanical Properties of Rubber Vulcanizates Filled with Silica

	parameters					
samples	SE100 (MPa)	SE200 (MPa)	SE300 (MPa)	TS (MPa)	*E*_B_ (%)	*H* (°Sh A)
NR/SiO_2_	0.9 ± 0.1	1.5 ± 0.1	2.4 ± 0.1	7.7 ± 0.7	598 ± 34	41.6 ± 0.3
NR/SiO_2__aminosilane	1.6 ± 0.1	3.3 ± 0.2	6.0 ± 0.3	7.6 ± 0.7	347 ± 11	45.4 ± 0.6
NR/SiO_2__mercaptosilane	1.1 ± 0.1	2.0 ± 0.1	3.4 ± 0.2	9.4 ± 1.0	461 ± 27	37.3 ± 0.3
NR/SiO_2__l-cysteine	0.9 ± 0.1	1.6 ± 0.1	2.7 ± 0.1	8.8 ± 0.7	571 ± 24	39.3 ± 0.1

Vulcanizates filled with surface-modified silica using l-cysteine, as a result of its oxidation to cystine,^63^ compared to vulcanizates containing native silica, are characterized by the deagglomeration of filler particles, separated by amino acid molecules adsorbed on the surface of silica particles according to the mechanism proposed in Figure 9.

**Figure 9:**
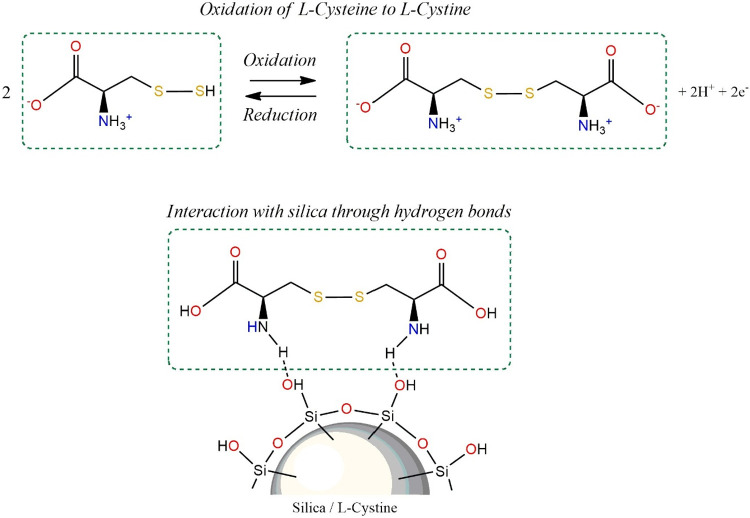
Mechanism of adsorption of l-cystine molecules on the surface of silica particles.

The FT-IR spectrum of SiO_2__l-cysteine (Figure 1a) shows distinct bands at 2580 cm^–1^, assigned to S–H stretching vibrations, indicating that the thiol group remains free and does not participate in covalent bonding with silanol groups. This excludes the formation of Si–S linkages.

The band at 1483 cm^–1^ (Figure 1c) is attributed to amino groups strongly hydrogen-bonded to surface silanols (Si–OH), suggesting specific NH···O–Si interactions. Additionally, the intensified double band at 1628 and 1584 cm^–1^, assigned to asymmetric COO^–^ stretching vibrations, indicates that the carboxyl group is predominantly present in its deprotonated form and may interact electrostatically with partially deprotonated silanols (Si–O^–^).

Importantly, no new bands characteristic of Si–O–C ester linkages (typically 1735–1750 cm^–1^) are observed, which excludes covalent ester formation between carboxyl groups and the silica surface.

The solid-state ^29^Si NMR spectra (Figure 2) further support this interpretation. For silane-modified samples, the presence of T^2^ and T^3^ structures confirms the formation of a covalent Si–O–Si–R bond (chemisorption). In contrast, such T-type signals are absent in the SiO_2__l-cysteine sample, indicating that cysteine does not form siloxane bonds with the surface.

Therefore, the interaction mechanism of l-cysteine with silica is predominantly noncovalent and involves

- hydrogen bonding between −NH_3_^+^/–NH_2_ and Si–OH,
- hydrogen bonding between carbonyl groups and silanols,
- electrostatic interactions between −NH_3_^+^ and Si–O^–^, and
- possible COO^–^···HO–Si interactions.

This adsorption layer reduces silanol–silanol hydrogen bonding between adjacent silica particles, leading to filler deagglomeration without increasing rubber–filler chemical coupling, which is consistent with the similar bound rubber content observed for NR/SiO_2_ and NR/SiO_2__l-cysteine compounds (Table 8).

Due to their polar nature, the side hydroxyl groups of l-cysteine molecules may potentially react with the polar silanol groups present on the surface of neighboring silica particles, contributing to the deagglomeration of the filler while not reacting with macromolecules of rubber. Hence, the designated contents of bound rubber in a silica-filled rubber mix with native silica and silica modified with l-cysteine are similar to each other. Table 8 does not show any significant difference.

Vulcanizates filled with silica modified by the aminosilane exhibit a similar Payne effect, compared to vulcanizates filled with silica modified with l-cysteine (Figure 8). However, contrary to the former, the grafting of the aminosilane molecules onto the surface of silica particles occurs through hydrogen bonds between the silanol groups on the surface of silica and hydroxyl groups in the 3-aminopropyltriethoxysilane molecules (Figure 10), in agreement with the literature.^64,65^

**Figure 10:**
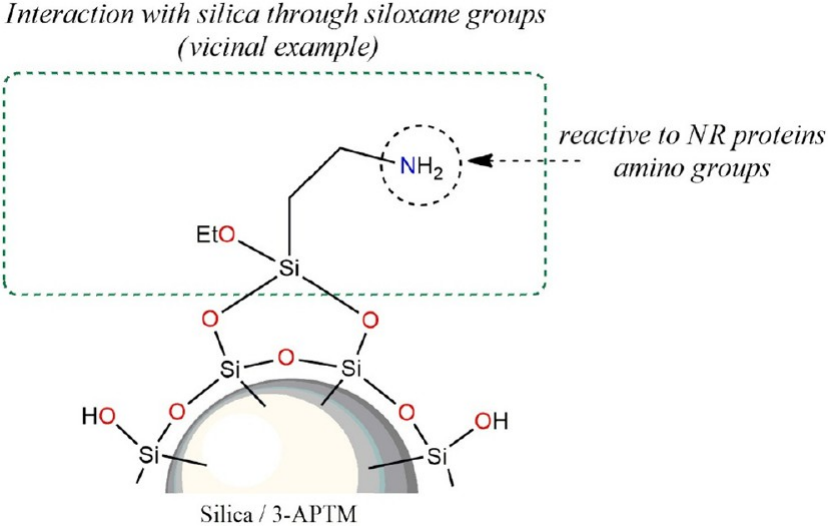
Mechanism of the adsorption of 3-aminopropyltriethoxysilane molecules on the surface of silica particles.

Considering the Payne effect, the result of silanization silica with the aminosilane is similar to that of l-cysteine (Figure 8) due to the fact that just as with l-cysteine, in this case, the modified filler particles are deagglomerated, but still exhibit a polar character, with the difference being that, in this case, there are amide groups that have a strong chemical affinity for the proteins present in natural rubber.^66^ Most likely for this reason, the rubber mixtures filled with silica modified with 3-aminopropyltriethoxysilane exhibited the highest content of bound rubber (Table 8).

The adsorption mechanism of the mercaptosilane molecules on the surface of silica particles is similar to that of aminosilane, proceeding through hydrogen bonds between silanol groups on the surface of silica and hydroxyl groups in the molecules of 3-mercaptopropyltrimethoxysilane, as presented in Figure 11.^13,52^

**Figure 11:**
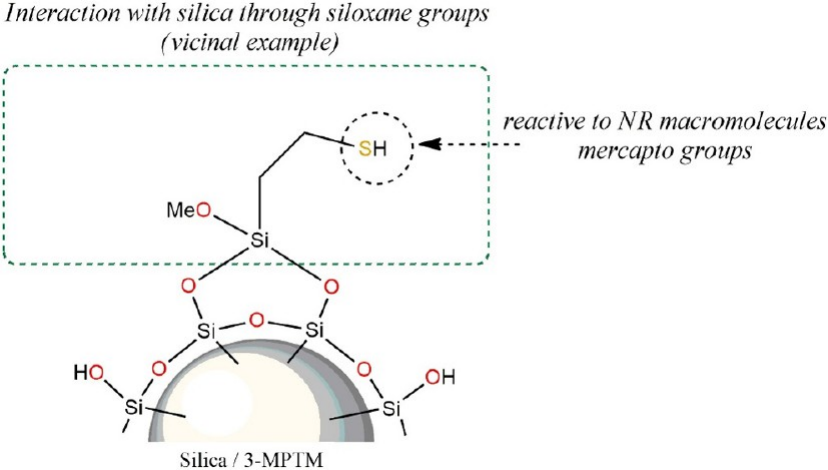
Mechanism of the adsorption of 3-mercaptopropyltrimethoxysilane molecules on the surface of silica particles.

Also, in this case, modification of the filler surface leads to its deagglomeration. The obtained effect is the largest among the studied modifications, as evidenced by the highest measured Payne effect (Figure 8). This can be explained by the reactivity of the thiol side groups of silane molecules, which chemically interact with the macromolecules of rubber,^67^ as confirmed by the highest measured value of bound rubber for the studied rubber blends (Table 8).

### 3.10 Dynamic Properties of Rubber Vulcanizates (DMTA)

The course of the tan δ (loss) of the rubber vulcanizates studied as a function of temperature is shown in Figure 12, and the value of the dynamic glass transition temperature (*T*_g_) along with the values of tan δ at characteristic points corresponding to *T*_g_ and responsible for slip/braking (0 °C) and rolling resistance (60 °C) are given in Table 11. It was expected that the above-mentioned values may change as a result of interphase filler–rubber or internal filler–filler interactions. However, a certain influence of the cross-link density or structure cannot be ruled out.

**Figure 12:**
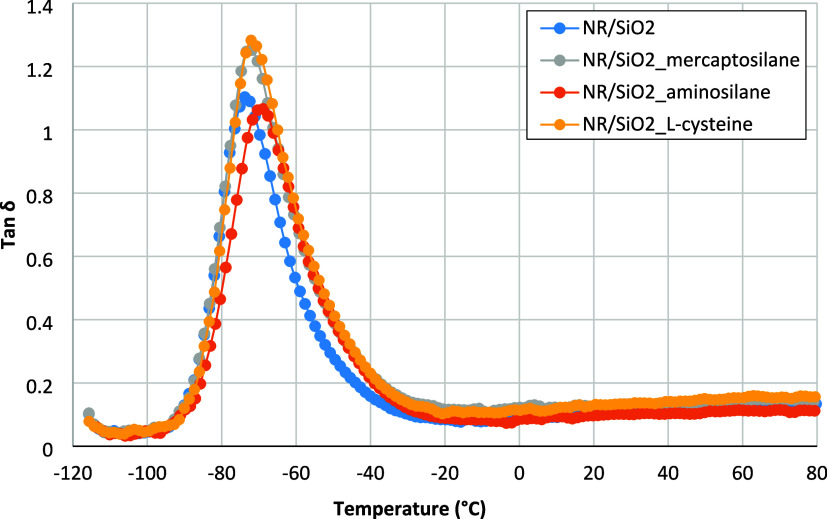
Course of the tan δ for the rubber vulcanizates filled with silica.

Modification of silica with mercaptosilane or l-cysteine slightly increases the glass transition temperature of vulcanizates containing it, while the use of aminosilane for this purpose makes the increase more significant. Also, the values of the tan δ of vulcanizates filled with the silica modified with mercaptosilane or l-cysteine are higher than those of vulcanizates filled with native silica or after surface modification with aminosilane. In light of the results obtained, the key factor determining the increase in the dynamic loss of filled vulcanizates is the dispersion of the filler in the rubber matrix. Vulcanizates containing silica modified with mercaptosilane or l-cysteine, which have better dispersion compared to NR systems with unmodified filler or with filler modified by aminosilane (Figure 7 and Table 8), exhibit higher tan δ values. Filler–rubber interactions seem to be less important. On the other hand, worse dispersion of the filler, combined, above all, with a higher cross-linking density in the case of NR filled with aminosilane-modified silica (Figure 6), is responsible for the significant increase in the glass transition temperature of the vulcanizates.

### 3.11 Mechanical Properties of Rubber Vulcanizates

The highest degree of cross-link density of the rubber vulcanizates filled with aminosilane-modified silica was accompanied by the smallest change in internal interactions in the filler phase, but the highest BdR content, which translates into the tensile strength of the rubber vulcanizates, which is the smallest in this case. The results of testing the mechanical properties under static conditions (ISO 37) of the rubber vulcanizates studied and determining their hardness are summarized in Table 12.

The obtained values of the tensile strength (TS) of silica-filled NR vulcanizates are not very high, but it is not unusual that they are lower in comparison to those of the unfilled rubber. The application of nanosized silica significantly decreases the cross-link density of NR vulcanizates, resulting in a significant reduction of TS.^16,68^ Nevertheless, the results of mechanical tests confirm the experimental data on the cross-link density and structure of the rubber vulcanizates. Apart from NR/SiO_2__aminosilane vulcanizates, all tested modifications improved the mechanical strength and stiffness of vulcanizates. The highest stiffness is observed for vulcanizates filled with silica modified with aminosilane, which is also reflected in their highest hardness. However, the highest strength was obtained for vulcanizates filled with silica modified with mercaptosilane, which correlates with their most diverse structure of cross-links and is reflected in the lowest hardness. In their case, the lowest share of polysulfidic cross-links was recorded. The slightly worse characteristics of vulcanizates filled with l-cysteine-modified silica in terms of strength may be a consequence of smaller interphase interactions and a higher elasticity, followed by the Payne effect.

## 4 Summary and Conclusions

The research confirms the influence of the modification of the silica surface on its microdispersion and interactions with the rubber matrix. The influence of modifications on the kinetics of vulcanization, cross-link density, and, to a small extent, cross-link structure of the rubber vulcanizates was also found. It is likely that the modification can also have some effect on the dispersion of the components of the curing system. All of the above effects explain the positive effects of the tested modifications on the mechanical and dynamic properties of natural rubber (NR) vulcanizates filled with modified silica.

The obtained results confirm that l-cysteine can be a substitute for the silanes used to modify silica, improving the dispersion but lowering the interaction with the rubber matrix in NR-based rubber mixtures.

The effect of an ex situ TESPT (Si69) treatment of silica on the filler dispersion in natural rubber (NR) and associated mechanical properties of sulfur-cured NR vulcanizates has been described recently by You and Jin.^69^ However, the successful modification, enhancing the mechanical properties of modified silica/NR composites, was granted only to the filler loading not exceeding 30 phr. Further incrementing the silane-modified SiO_2_ content for rubber reinforcement led to an uneven dispersion of the modified filler, thereby adversely affecting the overall performance of the composite material. This was not the case in our studies, where NR was loaded with 50 phr of modified silica. Additionally, the replacement of silane coupling agents with l-cysteine results in changes in cross-link density and structure, which, together with improved filler dispersion and unchanged BdR content, manifest themselves in the increase in the tensile strength of the rubber vulcanizates due to their significantly increased elongation at break and, contrary to silanization, observed decreased stiffness, especially in the case of mercaptosilane application. Despite the fact that the amino- and mercaptosilanes used are not conventional silanes used for NR systems, the general possibility of replacing silane compatibilizers (even TESPT) in rubber technology with cheap protein fragments, derived from recycled natural products like the thiol group-containing amino acids or protein hydrolyzates, can open a new direction of research toward bioelastomeric composites.
